# Premolar Extraction Affects Mandibular Kinematics

**DOI:** 10.1055/s-0042-1755629

**Published:** 2022-09-27

**Authors:** Alejandra Londoño, Miguel Assis, Cinzia Fornai, Markus Greven

**Affiliations:** 1 Department of Research in Occlusion Medicine, Vienna School of Interdisciplinary Dentistry — VieSID, Klosterneuburg, Austria; 2 Clinical Division of Prosthodontics, University Clinic of Dentistry Vienna, Medical University of Vienna, Vienna, Austria; 3 Institute of Evolutionary Medicine, University of Zurich, Zurich, Switzerland; 4 Department of Evolutionary Anthropology & Human Evolution and Archaeological Sciences (HEAS), University of Vienna, Vienna, Austria; 5 Center of Clinical Research, University Clinic of Dentistry Vienna, Medical University of Vienna, Vienna, Austria

**Keywords:** jaw tracking, axiography, premolar extraction, mandibular function, orthodontic treatment, temporomandibular disorders

## Abstract

**Objectives** The practice of premolar extraction in orthodontics is controversial for its potential detrimental effects on the stomatognathic system. However, the ways in which premolar extraction affects mandibular function are still poorly understood. The purpose of this study was to assess the influence of premolar extraction on mandibular kinematics by evaluating axiographic tracings.

**Materials and Methods** Forty-five orthodontically treated patients with premolar teeth extraction were compared with 45 paired untreated controls, selected for the absence of malocclusions. Systematic three-dimensional axiographic recordings of the mandibular movements were performed for protrusive–retrusive movements and speech. The transversal deviations and length of the movements were recorded for both sides along with the rotation angle during speech.

**Statistical Analysis** Differences between the axiographic variables were analyzed via the permutation test and Wilcoxon rank-sum test. Linear regression was performed to test whether axiographic parameters were predictive of group affiliation. Dot plots were used to explore the distribution of each of the axiographic outcomes, and isometric principal component analysis to assess the differences between the cumulative effects of premolar extraction on jaw motion.

**Results** The mandibular lateral translation in protrusion–retrusion and speech, the amount of rotation as well as the length of mandibular movements during speech were significantly higher in the treated subjects than in the controls, while retral stability did not differ. The linear regression yielded significant results for the mandibular lateral translation in protrusion–retrusion. The isometric principal component analysis showed higher values of the axiographic variables for 11 out of 45 individuals in the study sample compared with the control group.

**Conclusions** Premolar extraction altered mandibular kinematics in at least 25% of the cases within our sample, and the transversal discrepancy between protrusive and retrusive tracings was even predictive of group affiliation. These results support the notion that the routine practice of premolar extraction as part of the orthodontic treatment should be discouraged. It is compelling to perform further studies to assess whether a disrupted kinematics of the mandible is associated to temporomandibular disorders.

## Introduction

Premolar extraction is a common treatment strategy in orthodontics. The rationale behind premolar extraction is the creation of space for the realignment of crowded teeth and especially for a camouflage treatment of class II malocclusion. 1 However, this practice has not been free from controversy and the debate on the convenience of premolar extraction is ongoing since the onset of orthodontics. Some researchers have claimed that extraction results in a more effective and stable treatment with less need for patient's compliance in subjects with severe antero-posterior discrepancies or arch space deficiency 2 do not represent a risk factor for temporomandibular joint (TMJ) disorders. 3 Others showed that the same rate of relapses and crowding after orthodontic treatment with or without premolar extraction treatment is to be expected. 4 5 6 In the beginning of the 20th century, Angle 7 and his followers maintained that treatment without extractions was preferable. Very soon, Case 8 counter-argued Angle's teaching recommending extraction in less than 10% of the cases. In the mid-1940s, Tweed 9 considerably changed this conservative approach. He believed that functional mechanical balance depended on the vertical position of the mandibular incisors with respect to the basal bone and saw first premolar extractions as necessary to achieve this condition. In the late 1950s, following observations on Australian Aborigine's dentition, Begg 10 recommended extraction of the four first permanent premolars and in some cases of the four first molars to resolve the tooth size to arch length deficiency common in industrial societies, characterized by low degrees of dental wear. This resulted in the therapeutic use of premolar extraction in up to 80% of the orthodontic cases. Since the 1960s, with the general advancement in the orthodontic science and devices, this tendency started to change back into a more conservative approach. 11

From the functional point of view, the effect of premolar extractions on TMJ dysfunction, which is most prevalent from late adolescence to late middle age (approximately, 18 to 64 years of age), 12 is still unclear, and contrary to the clinical practice perception, 13 the literature does not provide an univocal response to this question. Studies based on pain or function evaluation did not find a definite relationship between temporomandibular disorders and previous orthodontic treatment, including those with premolar extraction. 14 15 Conversely, others found pathologic condyle position, loss of vertical dimension and TMJ dysfunction 16 as well as poor dynamic 17 and static 18 occlusion in patients treated with premolar extractions. Moreover, Yoon et al 19 realized that occlusal contact area during mastication did not re-establish fully even 2 years after extraction of the four premolars. Nonetheless, premolar extraction is still considered a valid practice, 20 (but see Moon et al 21 for a different point of view) and it is recommended in case of length discrepancy higher than 5 mm. 22

At present, the association between orthodontic treatment with premolar extraction and mandibular functional disturbances is still a matter of discussion within the dental community and the role of premolar extraction in mandibular kinematics is poorly understood. However, the occurrence of changes in the patients' facial profile after premolar extraction has been demonstrated, 23 and it can be expected that alterations of shape and length of the maxillary dental arch influence mandibular movements. Since the effects of premolar extraction on mandibular kinematics have not been investigated so far, the objective of the present study was to test whether orthodontic treatment with first premolar extraction alters the mandibular movements. To explore this phenomenon, a retrospective case–control study was designed to compare mandibular movements in patients who underwent orthodontic premolar extraction with a control sample with complete dentition. The symmetric mandibular movements in protrusion–retrusion and speech were recorded by means of axiographic tracings and the axiographic parameters were statistically analyzed to test possible differences between patients with and without premolars.

## Materials and Methods

### Sample Composition

The study group **(** hereafter P4ex **)** consisted of 45 consecutive, orthodontic patients who attended the private dental office of the main investigator (A.L.) (Centro Empresarial 128, Bogota, Colombia). These patients had already completed orthodontic treatment with premolar extraction elsewhere. The control group **(** hereafter P4 **)** included 45 class I patients with no history of orthodontic treatment, attending the UniCIEO University Foundation in Bogota, Colombia. All subjects signed an informed consent authorizing the confidential use of their data for this research, which was accepted by the Ethic Committee of UniCIEO University Foundation under the Act number 58 with approval number 93. Inclusion criterion for the study group was previous orthodontic treatment with bilateral upper or upper and lower premolar extractions. Patients with unilateral premolar extraction or lower premolar extraction only were excluded. For the control group, the inclusion criteria were complete dentition up to the second molars, Angle class I molar relationship, and absence of malocclusions of the anterior teeth such as open bite, cross bite, or more than 4 mm of crowding per arch. Subjects who underwent previous orthodontic treatment, gnathological, orthopaedic, or surgical interventions were excluded. None of the study or control subjects reported muscle or TMJ pain during the anamnesis, nor showed limited mouth opening.

### Recording of the Mandibular Movements

The mandibular movements were monitored by means of axiographic tracings using Cadiax Diagnostic computerized axiograph and Gamma software 8.6 (GAMMA Medizinisch-wissenschaftliche Fortbildungs-GmbH, Klosterneuburg, Austria) and were recorded by the same highly experienced operator (A.L.) as indicated by the manufacturer. Reliability and reproducibility have been proven for condylographic techniques. 24

**Fig. 1:**
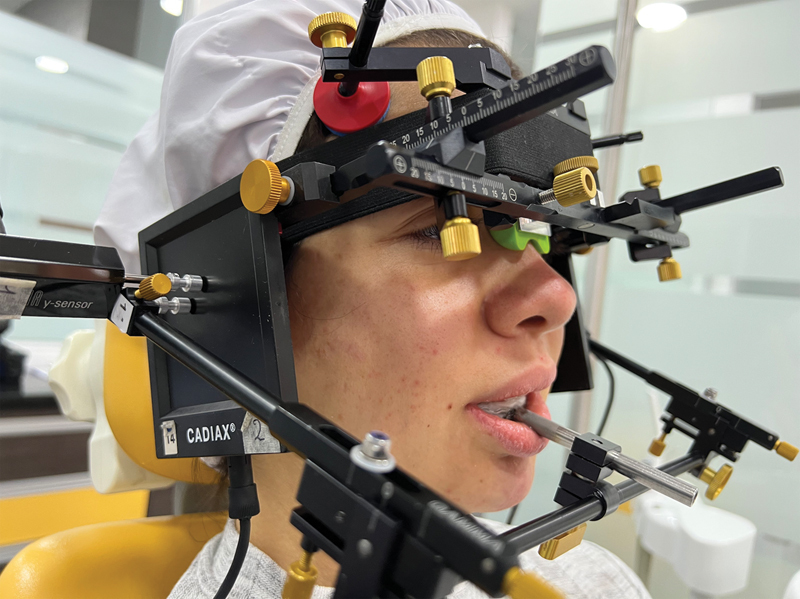
Photo of double electronic stylus in place, against the flag, kept in constant contact by magnetic tips, ready to measure the translation, rotation, and transversal components of the movement in each joint.

A mandibular face-bow with two double electronic styluses located close to the condyles was fixed to a clutch, bonded to the buccal face of the lower teeth to eliminate any dental interference during mandibular movements. An upper face-bow with vertical electronic plates on both sides registered the stylus movements, consisting of both translational and rotational components ( Fig. 1 ). The device allows also measuring the transversal component of the mandibular movements. The hinge-axis was first localized mechanically with paper and needle, and then it was fine-tuned with electronic aid. This procedure is mandatory before recording to avoid a distortion on the translation curves. The tracings were recorded with reference to the axis–orbital plane which is the plane determined by the hinge axis and the left orbitale, namely the lowest point on the orbit lower edge that can be felt under the patient's skin. Reference position (RP), 25 the starting point of every movement, was recorded after a 6 minute deprogramming of the masticatory muscles. The latter was achieved by positioning a cotton roll between the premolars, with the subject seated upright, with the head resting onto the support of the dental chair, keeping the teeth close together but free of dental contact. Then, the patient was asked to perform a few protrusion–retrusion movements, controlled by the operator with unforced chin-point guidance to feel the end of the retrusive path (which is called “end feel”). This allowed for the most accurate recording of the RP. Patients were instructed to perform free movements, with no guidance and no tooth contact, for maximal protrusion–retrusion and opening–closing movements. The movements were explained in detail and were rehearsed with the patient before recording. The standard movements were initiated in RP and were expected to end in RP unless there was lack of retral stability (see explanation for variable 5 below in this section). For the assessment of speech behavior, patients were asked to count backward from 70 to 60, as sibilant sounds are made in the most anterior and superior speaking positions. 26 The movements were performed at least three times to ensure that they were reproducible. These data could not be collected before premolar extraction, because the subjects of the study group were orthodontically treated at an earlier time, thus before they visited A.L.'s dental office.

**Fig. 2:**
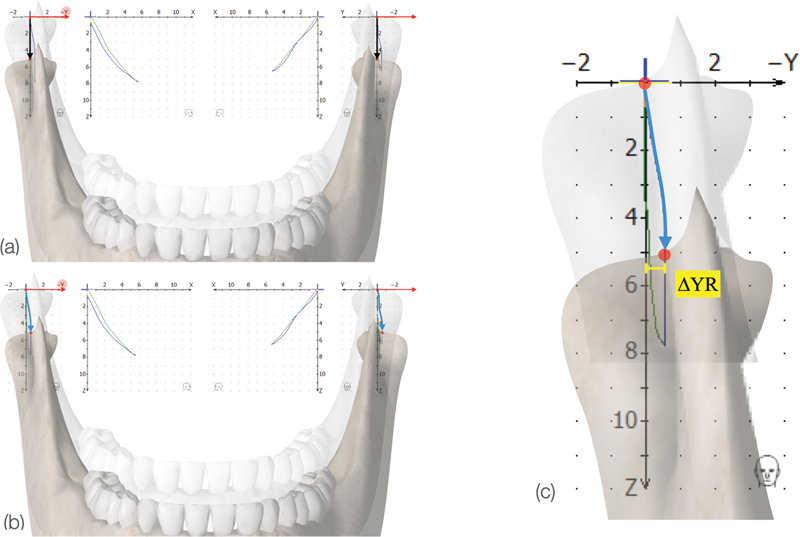
Protrusion/retrusion movement showing ( **a** ) normal movement and ( **b** ) mandibular lateral deviation on the *Y* -axis (∆YMLT) and ( **c** ) maximum mandibular lateral translation measurement on the axiographic tracing on the right side (∆YR).

Each movement was represented by a *X* – *Y* – *Z* coordinate matrix, where the *X* -axis was directed postero-anteriorly, the *Y* -axis was transversal and coinciding with the hinge-axis, and the *Z* -axis was vertical. The transversal movements of the condyles on the *Y* -axis were detected by the styluses attached to the mandibular face-bow. This mandibular lateral deviation, known as Delta Y and abbreviated here as ∆Y, consists of the maximum distance from the *Z* -axis representing the transversal movement of the subjects' tracings ( Fig. 2 ). This component of the mandibular movement is particularly important because it reflects deviations from supposedly symmetric movements.

**Fig. 3:**
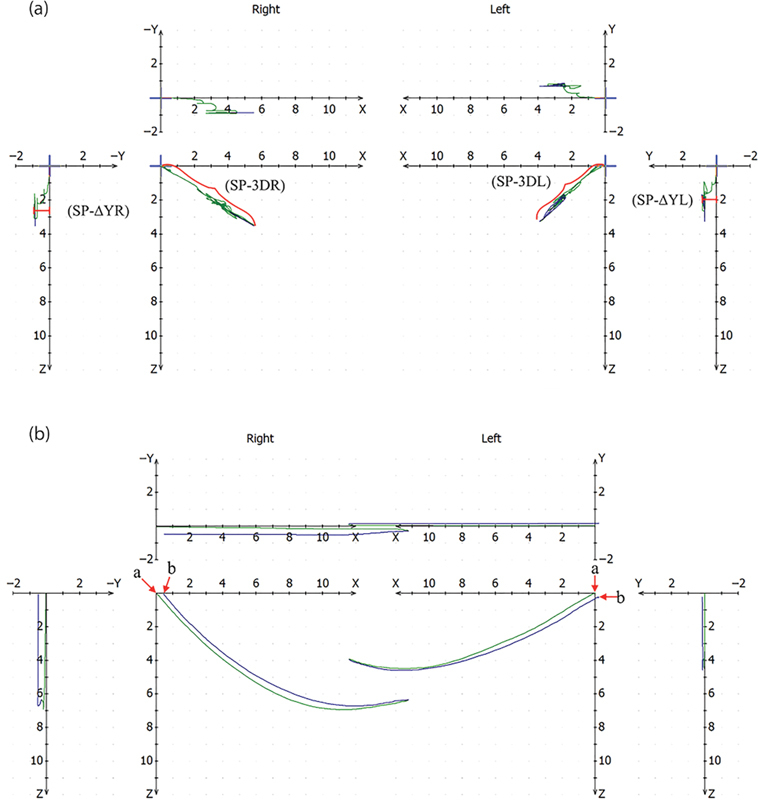
Axiographic tracings. ( **a** ) Diagram showing the maximum mandibular lateral translation (SP-∆Y) and the maximum length on the sagittal plane (SP-3D) on the right and left sides during speech. ( **b** ) Diagram showing retral stability as the distance in mm from the reference position (a *red arrow* “a”) and the end of the incursive movement ( *red arrow* “b”) during opening/closing movements.

The following five variables, of which four bilateral, were collected for both groups: (1) maximum perpendicular linear distance from the *Z* -axis of the protrusion–retrusion tracings representing lateral translation on the right and left sides (∆YR and ∆YL, respectively) ( Fig. 2C ); (2) length of the tracings projected onto the sagittal plane representing the protrusive excursion during speech for the right (SP-3DR) and left (SP-3DL) sides ( Fig. 3A ); (3) maximum amount of rotation along the *Y* -axis during speech (SP-G); (4) maximum perpendicular linear distance from the *Z* -axis of the speech tracings representing lateral translation on the right (SP-∆YR) and left (SP-∆YL) sides ( Fig. 3A ); (5) distance between RP and the end point of incursion in closing movements, or retral stability, on the right (R-STR) and left side (R-STL) ( Fig. 3B ). These measurements were gathered directly from the software, placing a cursor along the tracings and recording the relevant values. Distances were expressed in mm and rotation in degrees.

### Statistical Analyses

To assess the intra-operator error, 10 patients among the sample were randomly selected, and the measurements were repeated after 4 weeks from the first data collection. The Dahlberg formula was used to calculate the intra-operator error of measurement. 27 The Gamma software computes the angular measurement of maximum rotation during speech automatically; therefore, calculating the intra-observer error for these measurements was unnecessary.

The descriptive statistical analysis was performed using Minitab Statistical Software. The Anderson–Darling test was applied to test for normal distribution of the axiographic data. The correlation between the data from the right and left sides was tested to check whether the set of variables could be reduced. Given the nonnormality of the data, which is truncated at 0, the group mean differences were calculated by means of a permutation test ( *N* = 1,000). The Wilcoxon rank-sum test was applied to test also for differences in the groups' distribution. Furthermore, a linear regression analysis was performed to assess whether the variables gathered were good predictors for group affiliation. The range of distribution of the various variables was shown by group using dot plots. Afterward, an isometric principal component analysis 28 was performed to observe the additive effect of the variables considered for the P4ex and P4 groups. For this purpose, the data need to be normalized if the different variables show diverse ranges of distribution. These analyses were performed in R statistical software ( www.R-project.org ). 29

## Results

The age of the P4ex group ranged from 16 to 57 years (mean age: 36.91 ± 11.2 years). The age of the P4 group ranged from 14 to 50 years (mean age: 27.95 ± 10.01 years). The distribution by sex was 63% females and 27% males in the P4ex group and 67% females and 33% males in the P4 group. The average intra-observer error for the linear measurements was 0.2 mm, which was considered clinically irrelevant. The descriptive statistics for the axiographic parameters including average values, standard deviations, and minimum and maximum values are shown in Table 1 . A high correlation was found between the right and the left side of the bilateral measurements ( Table 2 ), which allowed us to reduce the number of variables from nine (i.e., ∆YR, ∆YL, SP-3DR, SP-3DL, SP-G, SP-∆YR, SP-∆YL, R-STR, R-STL) to five (i.e., ∆Y, SP-3D, SP-G, SP-∆Y, R-ST) by averaging the values for the right and left sides of each bilateral variable.

**Table 1:** Descriptive statistics for the axiographic variables including mean, standard deviation, and range of values for the study group with premolar extraction (P4ex) and the control group with complete dentition (P4)

Groups		P4ex ( *n* = 45)		P4 ( *n* = 45)	
Axiographic movements		Mean (SD)	Range	Mean (SD)	Range
Protrusion/retrusion	∆YR	0.76 (0.011)	0.08–2.35	0.39 (0.015)	0.03–1.98
	∆YL	0.73 (0.008)	0.10–2.35	0.37 (0.16)	0.01–1.68
Speech	SP-3DR	2.78 (0.051)	0.20–9.28	1.82 (0.035)	0.14–7.49
	SP-3DL	2.54 (0.047)	0.29–9.78	1.70 (0.051)	0.13–7.57
	SP-G	3.60 (0.052)	0.26–12.29	2.84 (0.014)	1.08–6.98
	SP-∆YR	0.29 (0.008)	0.02–1.47	0.16 (0.005)	0.02–1.47
	SP-∆YL	0.24 (0.010)	0.02–1.49	0.15 (0.004)	0.02–1.47
Open/close	R-STR	0.39 (0.019)	0.03–2.70	0.30 (0.007)	0.04–2.25
	R-STL	0.35 (0.016)	0.02–4.04	0.34 (0.007)	0.04–2.17

Abbreviations: ∆Y, lateral translation between the protrusion and retrusion tracings; L, left side; R, right side; R-ST, distance between RP and the end point of incursion in closing movements, or retral stability;; SP-3D, protrusive excursion during speech; SP-G, rotation along the *Y* -axis during speech; SP-∆Y, lateral translation during speech.

Note: Variables are reported in mm, except for SP-G which is expressed in degrees.

**Table 2:** Outcomes of the correlation test between the right (R) and left (L) side of each bilateral variable

	∆YR	SP-3DR	SP-∆YR	R-STR
∆YL	0.92	–	–	–
SP-3DL	–	0.86	–	–
SP-∆YL	–	–	0.95	–
R-STL	–	–	–	0.70

Abbreviations: ∆Y, lateral translation between the protrusion and retrusion tracings; R-ST, distance between RP and the end point of incursion in closing movements, or retral stability; SP-3D, protrusive excursion during speech; SP-∆Y, lateral translation during speech.

The results of the permutation test and Wilcoxon rank-sum test are shown in Table 3 . The permutation test revealed significant differences between the P4ex and P4 group means for all variables (∆Y, *p* = 0.00199; SP-3D, *p* = 0.01698; SP-G, *p* = 0.02097; SP-∆Y, *p* = 0.01898) except for R-ST ( *p* = 0.24470). Even the Wilcoxon rank-sum test, a nonparametric test, showed that the P4ex group is significantly different from P4 for ∆Y ( *p* = 0.00055), SP-3D ( *p* = 0.01448), and SP-∆Y (0.00584), while, consistently with the permutation test, differences for R-ST were not significant ( *p* = 0.75908). Differently from the permutation analysis, the Wilcoxon rank-sum test showed a nonsignificant result for SP-G ( *p* = 0.08937). In sum, the P4ex group showed statistically higher values than the P4 group for the transversal deviation in protrusion–retrusion and speech, and for the rotational component and length of speech. Differences in retral stability between study and control groups were not supported statistically. The results of the linear regression were significant only for ∆Y with a correlation of −0.34 with P4 and P4ex ( *p* = 0.001) ( Tables 3 and 4 ).

**Table 3:** Outcomes of the permutation test (number of iterations = 1,000) and Wilcoxon rank-sum test between the reduced variables from the study and control groups

Test	∆Y	SP-3D	SP-G	SP-∆Y	R-ST
Permutation	**0.00199**	**0.01698**	**0.02097**	**0.01898**	0.24470
Wilcoxon rank-sum	**0.00055**	**0.01448**	0.08937	**0.00584**	0.75908

Abbreviations: ∆Y, lateral translation between the protrusion and retrusion tracings; R-ST, distance between RP and the end point of incursion in closing movements, or retral stability; SP-3D, protrusive excursion during speech; SP-G, rotation along the *Y* -axis during speech; SP-∆Y, lateral translation during speech.

Note: Significant results ( *p* ≤ 0.05) are in bold.

**Table 4:** Outcomes of the linear regression for all axiographic variables, and correlation of the only significant variable, ∆Y

	Value	Std. Error	t stat	*p* -Value
Intercept	2.00075	0.1248	16.0864	**0.0000**
∆Y	−0.3012	0.1038	−2.9016	**0.0047**
SP-3D	**−0.0479**	**0.0298**	−1.6067	0.1119
SP-G	**−0.0333**	**0.0227**	−1.4677	0.1459
SP-∆Y	**−0.0136**	0.2122	−0.0640	0.9491
R-ST	−0.0998	0.1032	−0.9666	0.3365

Abbreviations: ∆Y, lateral translation between the protrusion and retrusion tracings; R-ST, distance between RP and the end point of incursion in closing movements, or retral stability; SP-3D, protrusive excursion during speech; SP-G, rotation along the *Y* -axis during speech; SP-∆Y, lateral translation during speech.

Note: Significant results ( *p* ≤ 0.05) are in bold. Multiple R-squared: 0.2053, adjusted R-squared: 0.158. F-statistic: 4.34 on 5 and 84 degrees of freedom; the *p* -value is 0.00146. The correlation for the first variable ∆Y is: *t* = −3.4171, df = 88, *p* -value = **0.001** . Alternative hypothesis: coef is not equal to 0. Sample estimates: correlation **−0.3423** .

**Fig. 4:**
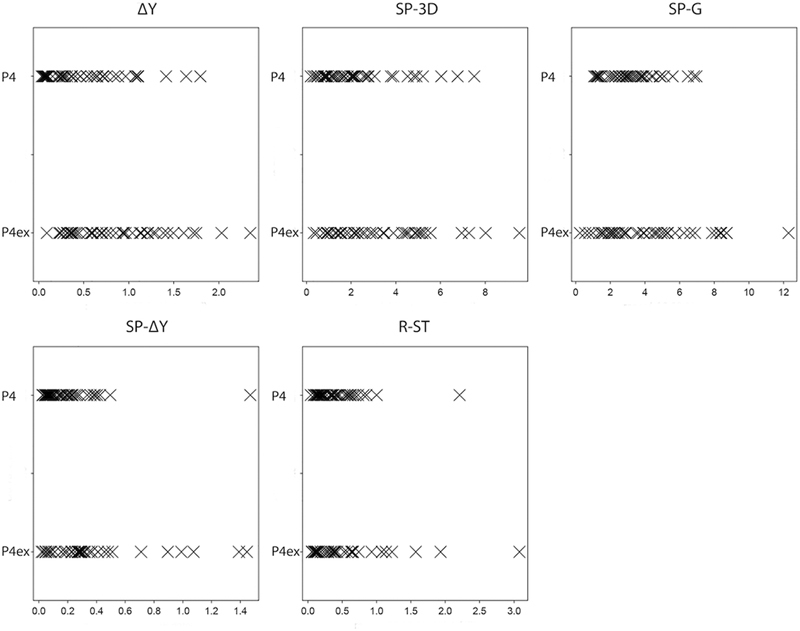
Dot plots (using crosses as symbols) showing the ranges of distribution of the various variable for the control group (P4, *upper rows* ) and for the study group (with premolar extraction, P4ex; *lower rows* ). ∆Y, distance from the reference position to the maximum point of lateral translation in protrusion/retrusion ; R-ST, distance between reference position and the end point of incursion in open/close movement; SP-3D, length of the movements performed during speech on the sagittal plane; SP-G, maximum rotation in degrees during speech; SP-∆Y, distance from reference position to the maximum point of mandibular lateral translation during speech.

**Fig. 5:**
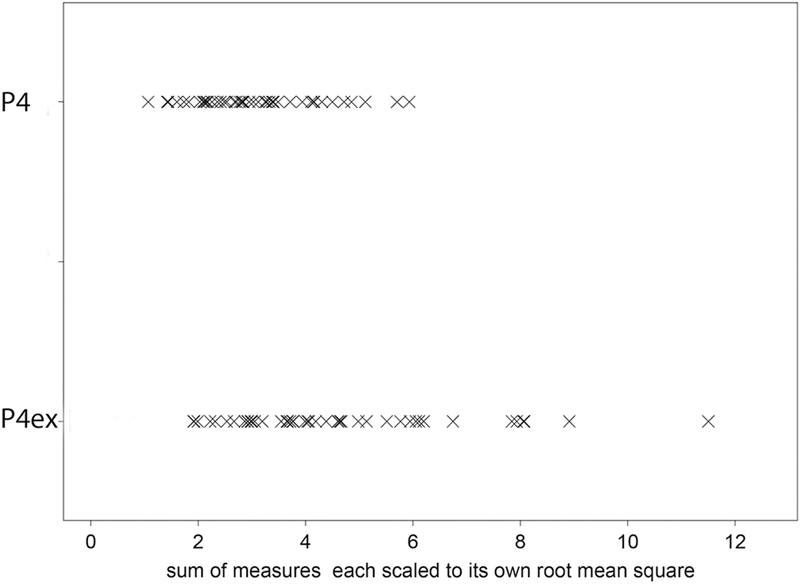
Isometric principal component analysis. *Upper row* : summary scores for the control group (P4); *lower row* : summary scores for the study group (with premolar extraction, P4ex).

The dot plots in Fig. 4 display the ranges of distribution by group for the various variables. The P4ex individuals presented the highest values of mandibular lateral translation in both protrusion–retrusion movements and speech in addition to longer tracings in speech with more rotational component. Since the maximum values for the various variables differed (∆Y = 2.35; SP-3D = 9.35; SP-G = 12.29; SP-∆Y = 1.47; R-ST = 3.08), a normalization by the root mean square around zero (i.e., squared root of the average of the squared values) was performed to plot the P4ex and P4 additive values ( Fig. 4 ), representing a summary of all variables. The additive values of the P4ex were higher than those of P4 in 11 out of 45 cases (25%) ( Fig. 5 ). The Wilcoxon rank-sum test performed on the additive values revealed a highly significant difference between the P4ex and P4 groups ( *p* = 0.00009).

## Discussion

The purpose of the present study was to identify possible differences in mandibular movements in patients orthodontically treated with premolar extraction, compared with untreated controls by means of computerized axiography. Computerized axiography and jaw tracking systems provide an objective view on the mandibular kinematics in three dimensions, thanks to their accuracy and reproducibility. 30

Notably, the results of the current study demonstrated that premolar extraction alters significantly mandibular kinematics, at least in 25% of the cases. We interpret this outcome as conservative, since our control group was selected only on the bases of the dental occlusion (presence of all premolars, class I relationship, absence of crowding, and deviations of the anterior dentition) while the absence of severe functional limitations was ascertained based on the anamnesis. However, we cannot rule out that the control group presented undetected, mild functional disturbances, which would make our results even more striking.

Mandibular lateral translation in protrusion–retrusion and speech, and the amount of rotation as well as length of speech were significantly higher in treated subjects than in controls, and lateral translation in protrusion–retrusion represented even a good predictor for belonging to the premolar extraction group.

To the best of our knowledge, the research by Heiser et al 6 is the only work previously published on the same topic treated here. These authors compared mechanic axiographic tracings for protrusion/retrusion movements in patients treated with dental extractions to a control group without extractions. They recorded the tracings before and soon after active orthodontic treatment and then again after the retention period and did not find significant differences between groups. The variables gathered by Heiser et al 6 are not directly comparable to those used in this study, because, contrary to us, they did not document the movements based on the hinge axis owing to technical limitations of the axiography device they used. However, they observed a significantly increased horizontal condylar inclination on the sagittal plane during protrusive movements after the retention period, in both groups. The authors interpreted this result as the outcome of normal growth process of the patients since the observations were made 3 years from base line during puberty.

Our findings are of major importance since transversal alterations of the mandibular movements (lateral translation along the *Y* -axis or ∆Y-MLT) have been related to disjunction of the condyle-disc complex and, in some cases, with disc displacement with reduction. 30 In a Master's thesis, 31 an average of 0.77 mm of mandibular lateral translation during protrusion–retrusion movements was found in patients with disc displacement, measured by magnetic resonance and axiography. This value is comparable to the average of 0.74 mm in the patients treated with extractions found in the present study. The translational and rotational components of speech were assessed in the present study. Despite speech being one of the most important functions of the human stomatognathic system, condylar movements during speech have been surprisingly under-investigated. Based on the incisal point trajectory, some researchers found an association between speech movements and the size of the maxillary dental arch, 32 skeletal class, 33 and the incidence of temporomandibular disorders. 34 The outcomes of these studies are not directly comparable to ours since the incisal trajectories do not match the condylar trajectories and occlusal factors like premolar extraction or previous orthodontic treatment were not considered.

Akimoto et al 35 analyzed axiographic speech condylar movements in relation to the various skeletal classes and found a maximum rotation of 3° and a mandibular lateral deviation of 0.16 mm in subjects free from temporomandibular disorders. Interestingly, these values are comparable to the ones obtained for the control group (retaining all premolars) in the current investigation (2.84° of rotation and 0.16 mm of mandibular lateral translation). Therefore, based on this evidence we can tentatively postulate that these values reflect physiological deviations of the condyle movements in subjects free from structural alterations of the masticatory system. Further studies are needed to corroborate this assumption.

It is widely acknowledged that orthodontic treatment of malocclusions improves self-esteem and emotional and social well-being, enhancing the quality of life in children, adolescents, 36 and adults. 37 However, treatments using premolar extractions might result in a reduced possibility to enhance the width and perimeter of the upper arch, causing instead its shortening and narrowing. 38 39 Premolar extractions can also change the inclination and position of the dental guidance, which may have an influence on the kinematics of mandibular movements at large. 40 Alterations of the occlusal guidance have been demonstrated to cause avoidance patterns. 41 42 We interpret the increased amount of rotation (3.6°) and mandibular lateral translation (0.29 mm) in our study group with extraction of the first premolars as resulting from a possible avoidance pattern of the mandible induced by a narrowing of the upper dental arch. In this instance, the mandible must open more than it would otherwise, to avoid interferences with more retruded upper incisors or because of possible changes in the transversal inclination of the canines. Although we did not investigate the shape of the TMJ structures in this study, the information collected during the anamnesis did not raise suspicion that severe degenerative changes might have affected the individuals analyzed. Based on these observations, we can confidently interpret the increase in mandibular movement parameters as determined by the changes in the occlusal guidance introduced by the extraction of the premolars.

Shortcoming of the present study consists in the lack of pretreatment axiographic data for the patients with premolar extractions and the lack of control of the time lapse between treatment finalization and the moment of evaluation. These limitations could be solved in future longitudinal studies. The role of orthodontic treatment with premolar extractions, considering variables such as time and modality of the treatment, in the insurgence or perpetration of signs and symptoms of temporomandibular disorders needs further investigation.

## Conclusions

Orthodontic extraction of first premolars might disrupt jaw kinematics which is reflected in the increased values of the axiographic parameters describing the condylar trajectories during protrusive–retrusive movements and speech. Thus, extraction of the premolars introduces a permanent change into the patients' stomatognathic system that is not only structural but also functional, with mostly unknown medium- and long-term effects on the craniomandibular system. This evidence warns against extraction of premolars as part of the orthodontic treatment. If space is needed, the extraction of third molars can be considered a suitable alternative to premolar removal. The alterations of jaw kinematics might be put in relation to temporomandibular and speech disorders although further studies are needed to ascertain these possible associations. If this evidence will be confirmed and a definite relationship between premolar extraction and dysfunctions of the stomatognathic system will be proved, the orthodontic extraction of premolars must be discouraged. Additionally, the evaluation of mandibular movements should be recommended as an integral part of diagnostic and follow-up assessment of treatment outcome.
